# Association of urinary monomethylated arsenic concentration and risk of hypertension: a cross-sectional study from arsenic contaminated areas in northwestern China

**DOI:** 10.1186/1476-069X-12-37

**Published:** 2013-04-21

**Authors:** Xin Li, Bing Li, Shuhua Xi, Quanmei Zheng, Da Wang, Guifan Sun

**Affiliations:** 1 Department of Environmental and Occupational Health, School of Public Health, China Medical University, No. 92 BeiEr Road, Heping District, Shenyang, Liaoning Province, 110001, China

**Keywords:** Urinary arsenic speciation, Monomethylated arsenic, Hypertension

## Abstract

**Background:**

Although some studies mainly from Taiwan, Bangladesh and the United States, have suggested a consistent dose–response increase in the prevalence of hypertension with increasing arsenic exposure, the association between chronic environmental arsenic exposure and the risk of hypertension is still inconclusive. Most of the studies discussed the association from the point of view of arsenic concentration in drinking water or cumulative arsenic exposure (CAE), few involved arsenic speciation into the discussion. In this cross-sectional study, we evaluated the potential association between environmental arsenic exposure through drinking water and the prevalence of hypertension by analyzing not only CAE but also urinary arsenic speciation, and provided data on arsenic exposure and hypertension from mainland of China.

**Methods:**

A cross-sectional study was conducted in one of the arsenic contaminated areas in the northwest of China. Among a total of 1005 residents who voluntarily participated in the study, 604 of eligible subjects were confirmed and interviewed door to door. Standing height, body weight, and blood pressure were measured. First void urine was collected and measured for the concentration of urinary arsenic speciation. CAE was calculated in a subpopulation of 360 subjects with detailed water consumption history. The association between urinary arsenic speciation, CAE and the risk of hypertension were analyzed by multiple logistic regressions.

**Results:**

We found that the levels of urinary arsenic species of inorganic arsenic (iAs), monomethylated arsenic (MMA), dimethylated arsenic (DMA) and total arsenic (tAs) were significantly correlated with systolic or pulse blood pressure. A positive relationship was found between the highest tertile of CAE and hypertension in a dose-dependent manner. Subjects with higher concentration of urinary MMA or lower percentage of DMA tended to be liable to suffer from hypertension. A significant increasing trend of the risk of hypertension with increasing tertiles of MMA concentration was also observed in the logistic regression models both before and after adjustment for confounders.

**Conclusions:**

Our findings suggested that arsenic exposure, especially high level of CAE, was positively associated with the prevalence of hypertension, and that higher concentration of urinary MMA might be related to the increased susceptibility to hypertension.

## Background

Arsenic is a toxic metalloid element which is present in the air, water and soil in both organic and inorganic forms. The main source of environmental exposure to inorganic arsenic (iAs) in humans is drinking water. The World Health Organization (WHO) recently changed the acceptable level of arsenic in drinking water to 0.01 mg/L. However, many countries have kept the standard of 0.05 mg/L which was established in 1975. Among the environmental exposures, arsenic contaminated drinking water has been a major public health problem throughout the world [1,2]. Millions of people around the world, such as Bangladesh, China, India, Cambodia, Argentina and Mexico, are being exposed to high level of arsenic through drinking water and facing serious health hazard of arsenic-related diseases [3].

In human, iAs is mainly metabolized enzymatically and non-enzymatically in liver into monomethylated arsenic (MMA) and dimethylated arsenic (DMA), which are then rapidly excreted into the urine [4,5]. Methylation was previously considered as a detoxification mechanism [6], however, recent studies have identified that the intermediate metabolites of arsenic methylation, especially trivalent MMA (MMA^III^), are far more toxic than the inorganic arsenic compounds [7,8]. Urinary arsenic speciation of iAs, MMA, and DMA, is usually used as the indicator for arsenic metabolism [9].

The association between the increased prevalence of hypertension and the long-term arsenic exposure through drinking water was first reported among the residents living in the endemic arsenic poisoning villages of Taiwan in 1995 [10]. Then, epidemiological researches from Taiwan, Bangladesh and the United States continuously reported the evidences that individuals chronically exposed to arsenic through drinking water might be at higher risk of developing hypertension compared to people unexposed, and that the effects were dose-dependent [11-14]. Recently, a Meta-analysis on the association between chronic arsenic exposure through drinking water and the prevalence of hypertension which included 8 relevant cross-sectional and cohort studies suggested that existing human data provided limited support for an association between arsenic and hypertension. It is still inconclusive although arsenic is possibly associated with hypertension [15]. While another systematic review that evaluated the relationship between arsenic exposure and hypertension end points by Meta-analysis from 11 cross-sectional studies (including 2 studies not exposed to arsenic through drinking water) identified an association between arsenic exposure and the prevalence of hypertension, but found limited interpretations of a causal effect of environmental arsenic on hypertension [16]. Due to the widespread arsenic exposure through drinking water in the world, even a small possible association between arsenic and hypertension might lead to a large number of cases and heavy burden of diseases caused by hypertension. Apart from Taiwan, it is estimated that nearly millions of people are being exposed to high level of arsenic (beyond the national standard of 0.05 mg/L) through drinking water in the mainland of China, and tens of thousands of them are suffering from arsenic-related diseases [17]. However, data from China on the relationship between arsenic exposure through contaminated drinking water and hypertension is scarce. In this research, we conducted a cross-sectional study in a county of Shanxi province, one of the arsenic contaminated areas in China, to evaluate the potential association between environmental arsenic exposure through drinking water and the prevalence of hypertension by analyzing urinary arsenic speciation and cumulative arsenic exposure (CAE), with emphasis on the association between arsenic speciation and hypertension, which has been rarely discussed up to now.

## Methods

### Study subjects

This study was conducted in Shanyin county of Shanxi province, one of the serious arsenic contaminated areas found in the 1980s, which locates in the northwest of China. Drinking water supply system in this region was dependent on tube wells. Arsenic concentration ranged from 0 to 0.65 mg/L according to the report of local public health government. Since northwest of China is a multinational area, only residents of Han nationality (accounts for 80 percent of the residents) were recruited so as to decrease the potential effects of gene polymorphism on the metabolism of arsenic methylation. Besides, residents living in the study county more than 10 years were considered as eligible subjects. Residents who took seafood in the past one week before the study were excluded. Among a total of 1005 residents who voluntarily participated in the study, 604 of eligible subjects were confirmed, and interviewed door to door. Standardized personal interview based on a structured questionnaire were carried out by well-trained interviewers. Information obtained from the interview included water consumption history, life style of cigarette smoking and alcohol consumption, medical history, family history of hypertension, diabetes, and other cardiovascular diseases, and other information such as socioeconomic status, demographic characteristics, and dietary habit. Comprehensive physical examination of residents recruited was completed by experienced physicians. Standing height and body weight were measured with a rigid vertical height measurement and a standard medical balance scale, with subjects not wearing shoes and in light clothes. Both interviewers and physicians were blind to the arsenic concentration to which subjects were exposed until all the interviews and physical examinations were completed. This study was approved by the institutional committee of China Medical University, and followed by the procedures of the institutional guidelines. All of the residents who participated in the study read and signed informed consent.

### Blood pressure measurements and diagnosis of hypertension

According to the protocol recommended by the WHO [18], blood pressure was measured three times with a mercury sphygmomanometer in sitting position after rest and relaxation for at least 15 minutes. Systolic blood pressure and diastolic blood pressure were defined at the first and fifth KorotKoff sounds, respectively. The lowest value was taken as the proper value for this study. Subjects who were found with elevated blood pressure were rechecked for another two measurements a few days later to validate the finding of hypertension (once per visit). Hypertension was defined in this study as a systolic blood pressure ≥ 140 mm Hg, a diastolic blood pressure ≥ 90 mm Hg, or a history of hypertension under regular treatment with antihypertensive agents.

### Arsenic exposure measurements

Among the total 604 eligible subjects, 360 subjects provided detailed residential history and arsenic concentration in the wells which they used in each of the residential duration. Since arsenic concentration was detected and reported to the family by the local public health government, it was considered as reliable in this study. CAE was calculated for each subject in the subpopulation with detailed water consumption history (*n* = 360). CAE in mg/L-year was defined as ∑(Ci×Di), where Ci is the arsenic concentration in mg/L in the tube wells which the subjects used in that residential duration, and Di in years is the duration of water consumption. CAE was not calculated in 244 out of the 604 subjects due to unclear arsenic concentration or obscure memories of the residential history.

### Urine collection and arsenic speciation measurements

The midstream of the first void urine was collected, kept on ice, immediately transferred to Centre for Disease Control and Prevention of Shanyin County, and stored at -20 centigrade. Samples then were transported on dry ice to the Laboratory of Arsenic Analysis in China Medical University, stored at -80 centigrade, and finally measured for urinary arsenic speciation within 3 months.

Measurement of arsenic speciation was performed as described previously [19]. Briefly, 1ml urine was digested with 2 M NaOH at 95 centigrade for 3 hours. The samples were stirred with a magnetic stirrer once every 30 minutes. Then the treated samples were diluted to 10 ml. iAs, MMA and DMA were measured by atomic absorption spectrophotometer (AA6800, Shimadzu, Japan) with an arsenic speciation pretreatment system (ASA-2SP, Shimadzu, Japan). Aliquot samples were used for each assay. Arsenic speciation was based on the well-established hydride generation of volatile arsines, followed by cryogenic separation in liquid nitrogen. The detection limit of each of the three arsenicals (iAs, MMA, DMA) was 1 ng, and the coefficient of variation was < 5%. The standard reference materials used were iAs standard of 1000 mg/L from the National Center for Standard Reference Materials (Beijing, China) and a mixed arsenic standard of 1000 mg/L MMA and DMA (Tri Chemical Laboratories Inc., Yamanashi, Japan). Quality control for arsenic measurement included the analysis of standard reference material of freeze-dried urine for toxic metals (SRM2670, National Institute of Standards and Technology [NIST], Gaithersburg, MD, USA). The NIST-certified concentration value for arsenic was 480 ± 100 μg/L. The value measured in our laboratory was 474 ± 20 μg/L. The value of each arsenic species was finally adjusted by the concentration of urinary creatinine (Cr). Total arsenic (tAs) concentration was calculated by summing the concentration of iAs, MMA and DMA. Urinary arsenic speciation was represented by urinary arsenic species (concentration of iAs, MMA, DMA and tAs in μg/g Cr) and urinary arsenic percentage (proportions of iAs, MMA and DMA, i.e. iAs%, MMA% and DMA%).

### Statistical analysis

Data analysis was carried out by SPSS software (version 13.0, SPSS Inc., Chicago, IL, USA). The differences of the categorical variables (gender, cigarette smoking and alcohol consumption) and continuous variables (age, body mass index (BMI), urinary arsenic species and percentage) of the subjects between cases with and without hypertension were analyzed by chi square test and Student’s *t* test, respectively. The relationship between urinary arsenic and blood pressure was analyzed by the Pearson correlation and partial correlation analysis. The associations between urinary arsenic speciation and hypertension, CAE and hypertension were analyzed by multiple logistic regression to estimate the multivariate adjusted odds ratios (OR) and 95% confidence intervals (CI) for hypertension risk.

## Results

### Characteristics of the study population

Totally, 604 subjects including 255 males and 349 females were included in this study. Characteristics of subjects with and without hypertension with regard to gender, cigarette smoking, alcohol consumption, age, BMI and urinary arsenic speciation were listed in Table 1. There were no differences between cases with and without hypertension in the distribution of gender, cigarette smoking and alcohol consumption. The average age, BMI, and urinary arsenic species of iAs, MMA, DMA and tAs of subjects with hypertension were significantly higher than those of the non-hypertension subjects, whereas urinary arsenic percentages of iAs, MMA and DMA were not significantly different between cases with and without hypertension.

**Table 1:** Characteristics of subjects with or without hypertension with regard to gender, cigarette smoking, alcohol consumption, age, BMI and urinary arsenic speciation

**Characteristics**	**Hypertension**		***p*-value**
	**No**	**Yes**	
***n***	436	168	
**Gender**^a^			
Male	182 (41.7%)	73 (43.5%)	0.703
Female	254 (58.3%)	95 (56.5%)	
**Cigarette smoking**^a^			
Never	293 (67.2%)	119 (70.8%)	0.390
Current or former	143 (32.8%)	49 (29.2%)	
**Alcohol consumption**^a^			
Never	336 (77.1%)	127 (75.6%)	0.702
Current or former	100 (22.9%)	41 (24.4%)	
**Age**^b^	46.37 (13.39)	57.58 (12.05)	<0.001
**BMI(kg/m**^**2**^**)**^b^	22.53 (3.19)	24.65(3.89)	<0.001
**Urinary arsenic species (μg/g Cr)**^c^			
iAs	12.83 (10.96, 15.02)	17.11 (13.44, 21.79)	0.050
MMA	17.35 (15.33, 19.62)	23.75 (19.33, 29.18)	0.009
DMA	94.17 (85.50, 103.70)	123.95 (105.26, 145.96)	0.004
tAs	135.59 (123.03, 149.43)	178.33 (151.43, 210.01)	0.004
**Urinary arsenic percentage (%)**^c^			
iAs	9.46 (8.62, 10.39)	9.60 (8.28, 11.12)	0.876
MMA	12.79 (12.06, 13.57)	13.32 (12.20, 14.55)	0.468
DMA	69.45 (67.52, 71.43)	69.51 (67.10, 72.00)	0.974

^a^ Values are number (%).

^b^ Values are mean (standard deviation).

^c^ Values are geometric mean (95% confidence interval).

### Relationship between urinary arsenic and blood pressure

As shown in Table 2, urinary arsenic species of MMA, DMA and tAs were positively correlated with systolic or pulse blood pressure with significant Pearson correlation coefficients. A significant positive correlation was also found between urinary arsenic specie of iAs and pulse blood pressure. No significant correlations were found between urinary arsenic species and diastolic blood pressure. Moreover, there were no significant correlations between urinary arsenic percentage and blood pressure (Table 2). After the adjustment for the variables of gender, age, cigarette smoking, alcohol consumption and BMI by partial correlation analysis, the results were similar with those of the Pearson correlation analysis as shown in Table 3. Urinary arsenic species of iAs, MMA, DMA and tAs were significantly correlated with the level of systolic or pulse blood pressure, while no significant correlations were found between urinary arsenic species and diastolic blood pressure, and between urinary arsenic percentage and blood pressure.

**Table 2:** Correlations between urinary arsenic and blood pressure by bivariate correlation analysis

	**Systolic blood pressure**		**Diastolic blood pressure**		**Pulse blood pressure**	
	**Pearson correlation coefficient**	***p*-value**	**Pearson correlation coefficient**	***p*-value**	**Pearson correlation coefficient**	***p*-value**
**Urinary arsenic species**						
iAs (μg/g Cr)	0.078	0.056	−0.015	0.706	0.130	0.001
MMA (μg/g Cr)	0.128	0.002	0.016	0.689	0.177	0.000
DMA (μg/g Cr)	0.157	0.000	0.034	0.400	0.204	0.000
tAs (μg/g Cr)	0.152	0.000	0.027	0.510	0.203	0.000
**Urinary arsenic percentage**						
iAs(%)	−0.032	0.436	−0.054	0.182	0.002	0.971
MMA(%)	0.017	0.673	−0.011	0.793	0.035	0.391
DMA(%)	0.016	0.699	0.027	0.507	−0.001	0.979

Data were based on log-transformed values.

**Table 3:** Correlations between urinary arsenic and blood pressure by partial correlation analysis

	**Systolic blood pressure**		**Diastolic blood pressure**		**Pulse blood pressure**	
	**Partial correlation coefficient**	***p*-value**	**Partial correlation coefficient**	***p*-value**	**Partial correlation coefficient**	***p*-value**
**Urinary arsenic species**						
iAs (μg/g Cr)	0.099	0.016	−0.017	0.682	0.158	0.000
MMA (μg/g Cr)	0.098	0.016	−0.009	0.827	0.149	0.000
DMA (μg/g Cr)	0.115	0.005	−0.001	0.984	0.166	0.000
tAs (μg/g Cr)	0.117	0.004	−0.007	0.868	0.176	0.000
**Urinary arsenic percentage**						
iAs(%)	0.041	0.311	−0.021	0.605	0.081	0.490
MMA(%)	0.011	0.796	−0.008	0.849	0.022	0.585
DMA(%)	−0.013	0.747	0.022	0.585	−0.040	0.327

Data were based on log-transformed values.

Correlations were adjusted by gender, age, cigarette smoking, alcohol consumption and BMI.

It is obvious that the more consumption of arsenic-contaminated water, the higher levels of urinary arsenic species. The findings of the correlations between urinary arsenic and blood pressure, together with the evidence that subjects with hypertension had significantly higher levels of urinary arsenic species of iAs, MMA, DMA and tAs than those without hypertension (Table 1) indicated that higher levels of urinary arsenic species might be associated with the elevated blood pressure, especially systolic and pulse blood pressure.

### Association between urinary arsenic speciation and the risk of hypertension

The results of the association between urinary arsenic speciation and hypertension by multiple logistic regression analysis were listed in Table 4. Subjects in the highest tertile of urinary MMA concentration showed a nearly 1.7 fold higher risk of hypertension than those in the lowest tertile before the adjustment for the potential confounders (OR: 1.776, 95% CI: 1.148, 2.746). There was a significant trend of increasing risk of hypertension in corresponding to increasing level of urinary MMA concentration (*p* = 0.009). The significant association between urinary MMA concentration and hypertension in the highest tertile, and the notable trend of increasing risk of hypertension in corresponding to increasing level of urinary MMA concentration still existed after the adjustment for the confounders. The OR (95% CI) of urinary MMA concentration for the risk of hypertension was 1.693 (1.028, 2.787). The *p* value of the trend was 0.033. The risk of hypertension was significantly higher in the highest tertile of DMA concentration when compared with that in the lowest tertile before the adjustment (OR: 1.812, 95% CI: 1.166, 2.817), with a notable trend of increasing hypertension risk along with increasing level of urinary DMA concentration (*p* = 0.008). After the adjustment, neither the association between urinary DMA concentration and hypertension, nor the trend of increasing hypertension risk along with increasing level of urinary DMA concentration existed. Subjects in the highest tertile of urinary tAs concentration carried a significant higher risk of hypertension compared to those in the lowest tertile before the adjustment (OR: 1.893, 95% CI: 1.219, 2.940), with a significant trend of increasing hypertension risk along with increasing level of urinary tAs concentration (*p* = 0.004). After the adjustment for the confounders, although the association between urinary tAs concentration and hypertension was not significant (OR: 1.648, 95% CI: 0.999, 2.721), there was a borderline-significance of 0.051. Moreover, the trend of increasing hypertension risk along with increasing level of tAs concentration remained significant (*p* = 0.046). As for the urinary arsenic percentage, subjects in the middle tertile of DMA% showed a significant decreasing risk of hypertension compared to those in the lowest tertile both before (OR: 0.596, 95% CI: 0.380, 0.935) and after (OR: 0.548, 95% CI: 0.327, 0.920) the adjustment, which indicated the association between DMA% and the risk of hypertension.

**Table 4:** Crude and adjusted odds ratio (95%CI) of hypertension by urinary arsenic species and percentage

	**Tertile**			**Test for trend**
**Urinary arsenic species**				
**iAs (μg/g Cr)**	<7.31	7.31 to 33.68	>33.68	
Hypertension (no/yes)	150/51	148/54	138/63	
Crude OR (95% CI)	(1.0)	1.073 (0.688, 1.675)	1.343 (0.869, 2.076)	*p*=0.182
Adjusted OR (95% CI)	(1.0)	1.301 (0.772, 2.192)	1.591 (0.963, 2.628)	*p*=0.070
**MMA (μg/g Cr)**	<11.28	11.28 to 37.89	>37.89	
Hypertension (no/yes)	154/47	151/50	131/71	
Crude OR (95% CI)	(1.0)	1.085 (0.687, 1.714)	1.776 (1.148, 2.746)*	*p*=0.009
Adjusted OR (95% CI)	(1.0)	0.956 (0.565, 1.617)	1.693 (1.028, 2.787)*	*p*=0.033
**DMA (μg/g Cr)**	<66.70	66.70 to 181.85	>181.85	
Hypertension (no/yes)	156/45	148/54	132/69	
Crude OR (95% CI)	(1.0)	1.265 (0.802, 1.994)	1.812 (1.166, 2.817)*	*p*=0.008
Adjusted OR (95% CI)	(1.0)	1.118 (0.661, 1.891)	1.549 (0.938, 2.559)	*p*=0.082
**tAs (μg/g Cr)**	<93.77	93.77 to 250.61	>250.61	
Hypertension (no/yes)	156/45	150/52	130/71	
Crude OR (95% CI)	(1.0)	1.202 (0.760, 1.899)	1.893 (1.219, 2.940)*	*p*=0.004
Adjusted OR (95% CI)	(1.0)	1.085 (0.641, 1.837)	1.648 (0.999, 2.721)	*p*=0.046
**Urinary arsenic percentage**				
**iAs (%)**	<7.30	7.30 to 15.76	>15.76	
Hypertension (no/yes)	141/60	155/47	140/61	
Crude OR (95% CI)	(1.0)	0.713 (0.457, 1.112)	1.024 (0.669, 1.568)	*p*=0.911
Adjusted OR (95% CI)	(1.0)	1.099 (0.653, 1.853)	1.483 (0.897, 2.453)	*p*=0.125
**MMA (%)**	<11.85	11.85 to 16.41	>16.41	
Hypertension (no/yes)	141/60	152/50	143/58	
Crude OR (95% CI)	(1.0)	0.773 (0.498, 1.200)	0.953 (0.620, 1.464)	*p*=0.824
Adjusted OR (95% CI)	(1.0)	0.747 (0.446, 1.252)	0.985 (0.586, 1.655)	*p*=0.958
**DMA (%)**	<68.73	68.73 to 79.07	>79.07	
Hypertension (no/yes)	138/63	158/43	140/62	
Crude OR (95% CI)	(1.0)	0.596 (0.380, 0.935)*	0.970 (0.636, 1.480)	*p*=0.887
Adjusted OR (95% CI)	(1.0)	0.548 (0.327, 0.920)*	0.697 (0.420, 1.156)	*p*=0.152

Data were based on log-transformed values.

Cut points were determined by tertile of overall study participants.

Adjusted OR were odds ratio adjusted for gender, age, cigarette smoking, alcohol consumption and BMI.

* *p*<0.05.

### Association between CAE and the risk of hypertension

Logistic regression analysis of hypertension in the subpopulation of 360 subjects by CAE showed a positive association between CAE and hypertension (OR: 1.913, 95% CI: 1.147, 3.191, *p* = 0.013) in the univariate logistic model. After the adjustment for gender, age, cigarette smoking, alcohol consumption and BMI, although the association was not significant (OR: 1.752, 95% CI: 0.992, 3.096), there was a borderline-significance of 0.053 (Table 5). When CAE was divided into categories by cut point of tertile, significant association between CAE and hypertension was found in the highest tertile of CAE > 0.35 mg/L-year in both models before (OR: 1.858, 95% CI: 1.064, 3.244) and after (OR: 1.871, 95% CI :1.022, 3.424) adjustment, with significant trends of increasing hypertension risk in corresponding to increasing level of CAE (*p* value of trend before adjustment: 0.026, *p* value of trend after adjustment: 0.040) (Table 6).

**Table 5:** Logistic regression analysis of hypertension in the subpopulation by CAE

	**Hypertension**	
	**No**	**Yes**
***n***	256	104
**Model I OR (95%CI)**	1.913 (1.147, 3.191)	
**Model II OR (95%CI)**	1.752 (0.992, 3.096)	

Model I: Univariate logistic regression model.

Model II: Model was adjusted for gender, age, cigarette smoking, alcohol consumption and BMI.

**Table 6:** Crude and adjusted odds ratio (95% CI) of hypertension in the subpopulation by CAE category

	**Tertile of CAE**			**Test for trend**
	**I**	**II**	**III**	
**Hypertension**				
No	91	89	76	
Yes	29	30	45	
**CAE (mg/L-year)**	<0.10	0.10 to 0.35	>0.35	
**Crude OR (95% CI)**	(1.0)	1.058(0.587, 1.905)	1.858(1.064, 3.244)*	*p*=0.026
**Adjusted OR (95% CI)**	(1.0)	1.204(0.632, 2.292)	1.871(1.022, 3.424)*	*p*=0.040

Adjusted OR were odds ratio adjusted for gender, age, cigarette smoking, alcohol consumption and BMI.

* *p*<0.05.

## Discussion

Tens of millions of people worldwide have been chronically exposed to arsenic-contaminated drinking water with the arsenic concentrations exceeding the WHO recommended level of 0.01 mg/L. Chronic environmental exposure to arsenic through drinking water is reported to be possibly associated with the elevated risks of hypertension and other cardiovascular diseases [20-22]. Up to date, only a few cross-sectional and cohort studies, mainly from Taiwan, Bangladesh and the United States, have investigated the association between hypertension and chronic arsenic exposure, however, it is still inconclusive [15,16]. Most of the reports have discussed the association from the point of view of arsenic concentration in the drinking water or CAE, few involved arsenic speciation into the discussion. In this study, we evaluated the potential association between environmental arsenic exposure through drinking water and the prevalence of hypertension by analyzing not only CAE but also urinary arsenic speciation, and provided data on arsenic exposure and hypertension from mainland of China.

In this study, we did not find any significant differences of urinary arsenic percentage between subjects with and without hypertension. The urinary concentration of arsenic species of iAs, MMA, DMA and tAs of subjects with hypertension were significantly higher than those of the subjects without hypertension. Besides, significant positive correlations were found between the concentration of urinary arsenic species (iAs, MMA, DMA and tAs) and blood pressure (systolic and pulse blood pressure). However, no significant differences were found between urinary arsenic percentage and blood pressure. These results suggested that the urinary concentration of arsenic species was associated with the elevated blood pressure, which indicated the possible role of arsenic in the development of arsenic-induced hypertension.

Since the levels of urinary arsenic species were dependent on the cumulative body burden of arsenic exposure, the higher cumulative arsenic burden, the higher urinary arsenic species. Our further findings of the positive association between CAE and hypertension in the subjects of the highest tertile of CAE, and the positive trend of increasing risk of hypertension in corresponding to increasing level of CAE indicated that the cumulative arsenic burden, which was demonstrated by urinary arsenic species, was associated with hypertension, and that there was a strong linkage between high level of CAE and the risk of hypertension.

Among the urinary arsenic species of iAs, MMA, DMA and tAs, we found that subjects with higher urinary concentration of MMA tended to be liable to suffer from hypertension with a significant dose-dependent trend. Besides, subjects with lower percentage of urinary DMA also showed an elevated susceptibility to hypertension. Since DMA was the methylated metabolite of MMA, lower percentage of DMA implied less production of DMA from MMA, which resulted in MMA remaining unmethylated and accumulated.

MMA, the intermediate metabolites of arsenic methylation, is more reactive and toxic than the other arsenic species, especially MMA^III^. It has been reported that the urinary level of MMA^III^, which is the most toxic species among identified metabolites of iAs, may serve as an indicator to identify individuals with increased susceptibility to toxic and cancer-promoting effects of arsenicosis [23]. Many researches including our previous studies indicated that high concentration or percentage of MMA in the urine was associated with arsenic-related skin lesions and cancers [19,24-26]. Our findings in this study suggested that higher concentration of urinary MMA might be related to the increased susceptibility to hypertension, one of the noncancerous diseases of arsenicosis. The MMA detected in this study included both the trivalent and pentavalent forms. We couldn’t detect the single concentration of MMA^III^ in the urine due to the undeveloped analysis system in our laboratory. It is obvious that the higher concentration of MMA in the urine, the higher level of MMA^III^ produced during the methylation pathway. Therefore, concentration of urinary MMA could be considered as a marker of MMA^III^ converted, although it is not direct. Our findings are quite compatible with the Huang group’s study which showed that subjects with hypertension had a higher CAE and a higher percentage of urinary pentavalent MMA (MMA^V^) than the subjects without hypertension in the arsenicosis-hyperendemic areas in southwest of Taiwan where residents stopped drinking the artesian well water for 2 to 3 decades [12].

Since MMA^III^ is the most reactive and toxic metabolite of arsenicals, recent studies have focused on the role of it on the mechanisms of arsenic-related cancer and noncancerous diseases. It has been reported that MMA^III^ could induce smooth muscle dysfunction through the disturbance of Ca^2+^ regulation, which resulted in impaired vasoconstriction and aberrant blood pressure change [27]. Lim et al. further indicated that low concentrations of MMA^III^ at nanomolar level could potentiate the agonist-induced vasoconstriction through Rho-mediated Ca^2+^ sensitization, which was manifested *in vivo* as increased pressor responses leading to dysregulation of normal physiological hemodynamics [28]. Our pervious study also found that MMA^III^ induced endothelial nitric oxide synthase (eNOS) phosphorylation in the endothelial cells which seemed to be an adaptive response at the early stage of exposure, but finally acted as a potent inhibitor of eNOS leading to disruption of eNOS bioactivity which consequently resulted in decreased NO bioactivity [29]. All of these studies provided evidences for the role of MMA^III^ in arsenic-associated cardiovascular disease of hypertension.

Because the participants of this study were recruited from a rural county in which residents almost had the same occupation as farmers, similar socioeconomic status and lifestyles, the variation among subjects of these potential confounders is likely to be small. Whereas dietary habits, such as salt intake and dietary pattern, might have an effect on the result. The lack of data collection on salt intake, which has been considered as a potential cause of hypertension, was a limitation of this study. Since low intake of calcium, animal protein, folate, and fiber were reported to increase the susceptibility of arsenic-related injuries [30], and some vitamins, especially folic acid, was found to act as the co-factors in the methylation metabolism of arsenic leading to variation of arsenic speciation between individuals due to different intake value [31,32], absent data of nutrient intake might also be a limitation of this study.

Another limitation of this study might be the unidentified gene polymorphisms involved in the metabolism and clearance of arsenic. Polymorphisms in a number of genes, including GSTO1 and GSTO2 from glutathione-S-transferase (GST) family which use glutathione as a reducing agent to catalyze pentavalent arsenicals to trivalent forms, GSTP1, GSTZ1, GSTM1, and GSTT1 of GSTs family which involved in the xenobiotic metabolism and play a role in the cellular response mechanism against oxidative stress induced by arsenic, arsenic 3-methyltransferase (AS3MT) which use S-adenosyl-methionine as a methyl donor to produce MMA and DMA, and methylene-tetrahydrofolate reductase (MTHFR) of one-carbon metabolism pathway, have been reported to be associated with the variation in arsenic methylation capacity [33-35] and susceptibility to arsenic-related skin lesions [36-38] and bladder cancer [39-42]. Since the role of gene polymorphisms in arsenic-related hypertension was rarely discussed which still need further research, we could not completely exclude the possible relationship between polymorphisms of these genes and susceptibility to the arsenic-related hypertension.

## Conclusions

Taken together, our findings suggested that arsenic exposure, especially high level of CAE, was positively associated with the prevalence of hypertension, and that higher concentration of urinary MMA might be related to the increased susceptibility to hypertension. Further studies are needed to evaluate the effects of low level arsenic exposure from drinking water on hypertension, and the association in some special aspects such as nutritional deficiencies and gene polymorphisms.

## Abbreviations

iAs: inorganic arsenic; MMA: Monomethylated arsenic; DMA: Dimethylated arsenic; tAs: Total arsenic; MMAIII: Trivalent MMA; MMAV: Pentavalent MMA; CAE: Cumulative arsenic exposure; BMI: Body mass index; GST: Glutathione-S-transferase; AS3MT: Arsenic 3-methyltransferase; MTHFR: Methylene-tetrahydrofolate reductase.

## Competing interests

The authors declare that they have no competing interests.

## Authors’ contributions

XL, BL, SX, QZ and GS designed the study. GS is the leader of this study. XL, BL, SX, QZ, DW and GS did the field work. XL, SX, QZ and DW detected the levels of arsenic speciation in the urine. XL, BL, and SX performed statistical analysis. XL drafted the manuscript. All authors read and approved the final manuscript.
